# Enhancing the accumulation of eicosapentaenoic acid and docosahexaenoic acid in transgenic Camelina through the CRISPR‐Cas9 inactivation of the competing *FAE1* pathway

**DOI:** 10.1111/pbi.13876

**Published:** 2022-07-11

**Authors:** Lihua Han, Richard P. Haslam, Susana Silvestre, Chaofu Lu, Johnathan A. Napier

**Affiliations:** ^1^ Plant Sciences Department Rothamsted Research Harpenden Herts UK; ^2^ Department of Plant Sciences and Plant Pathology Montana State University Bozeman Montana USA

**Keywords:** metabolic engineering, Omega‐3, Camelina, seed oil, gene editing, field trials

Omega‐3 long‐chain polyunsaturated fatty acids (LC‐PUFAs), eicosapentaenoic acid (EPA; 20:5Δ^5,8,11,14,17^) and docosahexaenoic acid (DHA; 22:6Δ^4,7,10,13,16,19^) are now accepted as being essential components of a healthy, balanced diet (Napier *et al.,* 2019; West *et al.,* 2021). The wild capture fisheries that supply omega‐3 fatty acids are at their maximum levels of sustainable production; therefore, attempts to meet the growing demands of an increasing population depend on alternative sources of fish oils (Tocher *et al.,* 2019). *Camelina sativa* is an oilseed crop with high levels (>35%) of α‐linolenic acid (ALA; 18:3Δ^9,12,15^) and has reconstituted a biosynthetic pathway from ALA to synthesize both EPA and DHA in Camelina cv. Celine seeds by expressing heterologous desaturase and elongase genes, producing levels of EPA and DHA equivalent to those in marine fish oils, exemplified by the prototype line DHA2015.1 (abbreviated to DHA1) accumulating over 25% n‐3 LC‐PUFAs (Figures S1 and S2 (Petrie *et al.,* 2014; Ruiz‐Lopez *et al.,* 2014). Field trials of DHA1 in the UK, USA and Canada demonstrated the omega‐3 LC‐PUFAs trait was stable in distinct geographical locations and agricultural environments (Han *et al.,* 2020). In parallel, salmon feeding trials and human dietary studies using DHA1 seed oils both demonstrated that these transgenic plant‐derived oils could serve as effective replacements for marine‐derived fish oils (Betancor *et al.,* 2018; West *et al.,* 2021).

Based on our observation that ALA is the endogenous C18 precursor for seed omega‐3 LC‐PUFA production (Han *et al.,* 2020), we hypothesized that increasing the ALA pool can further enhance EPA/DHA accumulation in DHA1 Camelina. The DHA1 construct already contains a Δ12 desaturase to drive the flux of fatty acids into PUFA biosynthesis (Figures S1 and S2). However, as a less obvious approach, we proposed using gene‐edited *fae1* mutants of Camelina. Camelina *FAE1* functions in competition with endogenous FAD2 Δ12 desaturase (which desaturates OA to produce linoleic acid (LA; 18:2Δ^9,12^)) for C18 substrates, sequentially elongating oleic acid (OA; 18:1Δ^9^) to gondoic acid 20:1Δ^11^ and then erucic acid 22:1Δ^13^. Previously, Ozseyhan *et al*. (2018) used CRISPR/cas9 to disrupt all three homologues of *FAE1* present in Camelina cv. Suneson (allohexaploid). Since the expression of FAE1 is restricted to developing seeds, there is no impact of this mutation on other tissues. Within the seeds, ALA was increased from 36.9% in wildtype (WT) to 47.3% in *fae1* mutant—this is in addition to the complete ablation of erucic acid, a fatty acid, which is considered undesirable above a modest threshold (5% of total oil) in human foodstuffs. Pollen from the *fae1* GE mutant (previously selfed to segregate away from the CRISPR‐Cas9 transgene and associated DsRed marker) was crossed with the DHA1 line described in Han *et al*. (2020), and the resulting F1 hybrid seeds were sown in the greenhouse. Selected seeds with strong DsRed fluorescence (associated with DHA1; Figure S2) from individual F2 plants were dissected, and half the cotyledon tissue was used for fatty acid composition analysis by GC‐FID, the other half germinated on 1/2MS nutrition media. Based on gas chromatographic analysis of fatty acid methyl esters (FAMEs) data, only seeds showing a low 20:1Δ^11^ content (<1.0%; Ozseyhan *et al.,* 2018) were identified as the DHA1 and *fae1* homozygous line (designated DHA1x*fae1*). Seeds from F3 plants were then sown alongside parental lines and WT as replicated plots in the field at the Rothamsted Experimental Farm (Harpenden, UK) on 23 May 2019, as permitted by Consent 19/R8/01 from DEFRA. After harvesting on 18 September 2019, replicated seed FAMEs and TAG analysis were completed for the plots.

GC analysis (Figure 1a) confirmed showed that in field‐grown DHA1x*fae1* seeds, the accumulation of 20:1Δ^11^ is as low as 0.4%, consistent with the mutant background. Compared with parental line DHA1, the levels of palmitic acid (16:0) and stearic acid (18:0) were broadly the same, with the OA and LA levels slightly decreased by 1% and 2.5%, respectively, whereas the ALA level was increased from 17.0% in DHA1 to 21.5% DHA1x*fae1*. This confirmed that targeted mutagenesis by gene editing of the *FAE1* genes in the DHA1 genetic background did not affect the OA precursor metabolites but did alter the downstream desaturation pathway, as predicted. Analysis of the total C20+ n‐3 LC‐PUFAs in DHA1x*fae1* seeds showed little change in EPA compared with DHA1 and 9.1% compared with 9.3%. However, the DHA level was modestly increased, from 9.7% in DHA1 to 12.6% in DHA1x*fae1*. Eicosatetraenoic acid (ETA; 20:4Δ^8,11,14,17^) and docosapentaenoic acid (DPA; 22:5Δ^7,10,13,16,19^) levels were similarly enhanced, 1.3% and 1.5%, respectively. In sum, total C20+ n‐3 fatty acids contents (comprising ETA, EPA, DPA and DHA) were increased by 5.5% from 27.5% in DHA1 to 33.0% in DHA1x*fae1*. These data demonstrated that increasing the precursor ALA substrate can be an effective approach to boost n‐3 LC‐PUFAs production in the DHA1 line.

**Figure 1:**
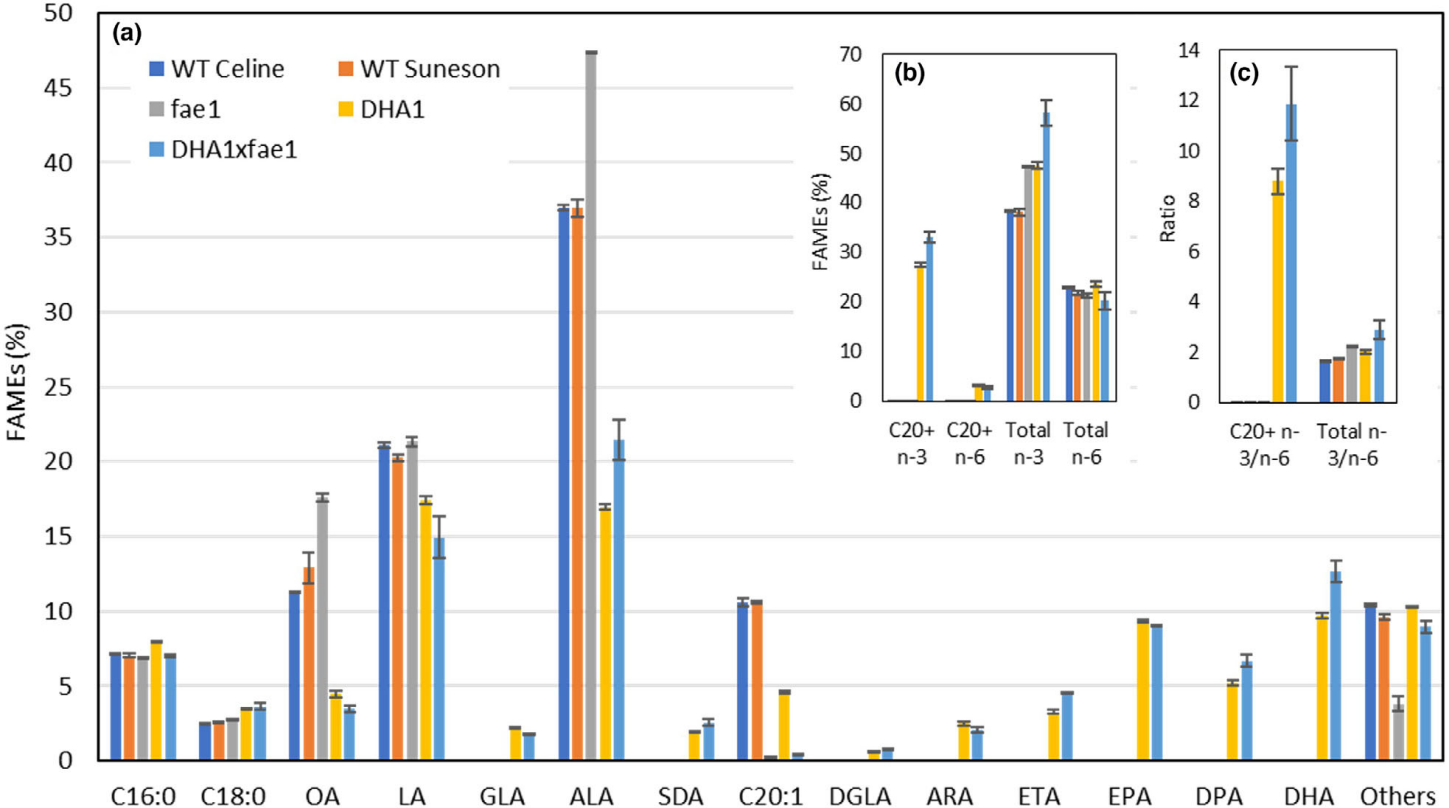
Omega‐3 long‐chain polyunsaturated fatty acids composition in Camelina sativa. Total seed fatty acids were analysed by GC‐FID after methanolysis. (a) Mol % fatty composition for WT, *fae1*, DHA1 or DHA1x*fae1* seeds. Note the absence of EPA and DHA in WT and *fae1* seeds, and how the levels generated by the DHA1 construct are elevated in the *fae1* background. (b) The % composition of omega‐3 (n‐3) and omega‐6 (n‐6) fatty acids. (c) The ratio of n‐3 to n‐6 fatty acids. [Colour figure can be viewed at wileyonlinelibrary.com]

Health studies in humans have reported that diets with an increased omega‐6 (n‐6) to n‐3 ratio are highly pro‐thrombotic and pro‐inflammatory, and contribute to the prevalence of atherosclerosis, obesity, diabetes and a wide range of inflammation disorders (West *et al.,* 2021). Therefore, we calculated the different n‐3 and n‐6 parameters (Figure 1b, c; Figure S3). The total C20+ n‐6 fatty acids including dihomo‐γ‐linolenic acid (DGLA; 20:3Δ^8,11,14^) and arachidonic acid (ARA; 20:4Δ^5,8,11,14^) remained similar, at 3.1% in DHA1 and 2.8% in DHA1x*fae1*, giving a ratio of C20+ (n‐3/n‐6) as 8.8 in DHA1 and 11.7 in DHA1x*fae1*. The total n‐3 content was 47.5% in DHA1 and 58.2% in DHA1x*fae1*, whereas the total n‐6 content was 23.6% and 20.2%, respectively. Therefore, the ratio of total (n‐3/n‐6) was 2.0 in DHA1 and 2.9 in DHA1x*fae1*, which is also a significant increase compared with 2.2 in the *fae1* mutant and 1.7 in both WT Celine and WT Suneson lines, indicating that DHA1x*fae1* seed fatty acids have a even better health benefits than those of DHA1, *fae1* and WTs.

Triacylglycerol (TAG) is the major storage lipid for fatty acids in the seed, and it consists of a glycerol backbone onto which three fatty acids are esterified. The fatty acid composition of individual TAG species (described by number of carbons:number of desaturations) can vary, and WT camelina seed can have ~80 different molecular species of TAG (Usher *et al.,* 2017). We have previously shown how transgene‐derived accumulation of EPA, DPA and DHA expands the repertoire of TAG species (Han *et al.,* 2020). A comparison (Figure S4) of the TAG species in the seeds of field‐grown DHA1 and DHA1x*fae1* compared to relevant parental lines (WT Celine, WT Suneson, *fae1*) illustrates a change in the TAG fingerprint of *fae1*, DHA1 and DHA1x*fae1*. The most abundant TAGs in fae1 are C54 species with 3 to 9 unsaturations—combinations of ALA (18:3) and LA (18:2). There is an almost complete absence of any C56 TAG species in *fae1*. In the case of DHA1 and DHA1x*fae1*, more novel TAG (C58 but inclusive of C56 to C66) species are present. These novel TAG species are absent from all the parental and WT backgrounds. Notable is the occurrence of some DHA‐specific TAG molecular species that differ between DHA1 and DHA1x*fae1* (e.g. 64:17 and 66:17) and a number of TAG species such as 58:8–58:12 that are more abundant, indicative of elevated accumulations of long‐chain polyunsaturated fatty acids.

In conclusion, the combination of CRISPR‐Cas9 gene editing to inactivate the FAE1 pathway clearly results in a beneficial increase in the levels of EPA, DHA and other omega‐3 LC‐PUFAs in transgenic Camelina harbouring the DHA2015.1 transgenes. In particular, the *fae1* mutant not only is devoid of C20+ monounsaturated fatty acids (including the undesirable C22 erucic acid; Bach and Faure, 2010) but also has increased levels of omega‐3 fatty acids such as ALA. Our previous studies have indicated that ALA is the primary endogenous precursor fatty acid used to make EPA and DHA, and our data here further confirm this. This contrasts with Canola, where recent attempts to engineer the accumulation of EPA and DHA result in the metabolism of the omega‐6 precursor LA. In that respect, Canola is biased towards the synthesis of omega‐6 fatty acids whereas Camelina is biased towards omega‐3, therefore Canola requires an additional transgene‐derived ‘push’ to direct the flux of fatty acids onto the omega‐3 track (discussed in Napier *et al.,* 2019). It is also noteworthy that Canola already lacks the FAE1 activity, selected by conventional plant breeding for the absence of erucic acid, which is present in parental varieties of *Brassica napus* seed oil. Collectively, these data suggest that Camelina is a superior host for the transgene‐derived seed‐specific synthesis of omega‐3 LC‐PUFAs such as EPA and DHA, and representing the first example of an environmental release of a GM + GE stack.

## Declaration of interest

JAN acts as a scientific advisor for Yield10 Biosciences. The other authors have no interests to declare.

## Author contributions

Experimental data were planned and collected by LH, SS, RH and CL. Constructs were designed and built by LH and JN. All authors contributed to the writing of this manuscript.

## Supporting information


**Figure S1** Schematic representation of biosynthetic pathways.
**Figure S2** Schematic representation of the DHA2015.1 construct.
**Figure S3** Unsaturation index of seed lipids.
**Figure S4** TAG profile for field‐grown material.
