# Supplementary material for: Enhancing the accumulation of eicosapentaenoic acid and docosahexaenoic acid in transgenic Camelina through the CRISPR‐Cas9 inactivation of the competing FAE1 pathway
Source: Plant Biotechnol J. 2022 Jul 11;20(8):1444–6. doi: 10.1111/pbi.13876 (PMC9342609; doi:10.1111/pbi.13876)
Supplement: Supplementary file 1 — Figure S1 Schematic representation of biosynthetic pathways. Figure S2 Schematic representation of the DHA2015.1 construct. Figure S3 Unsaturation index of seed lipids. Figure S4 TAG profile for field‐grown material. [file PBI-20-1444-s001.pptx]

## Slide 1
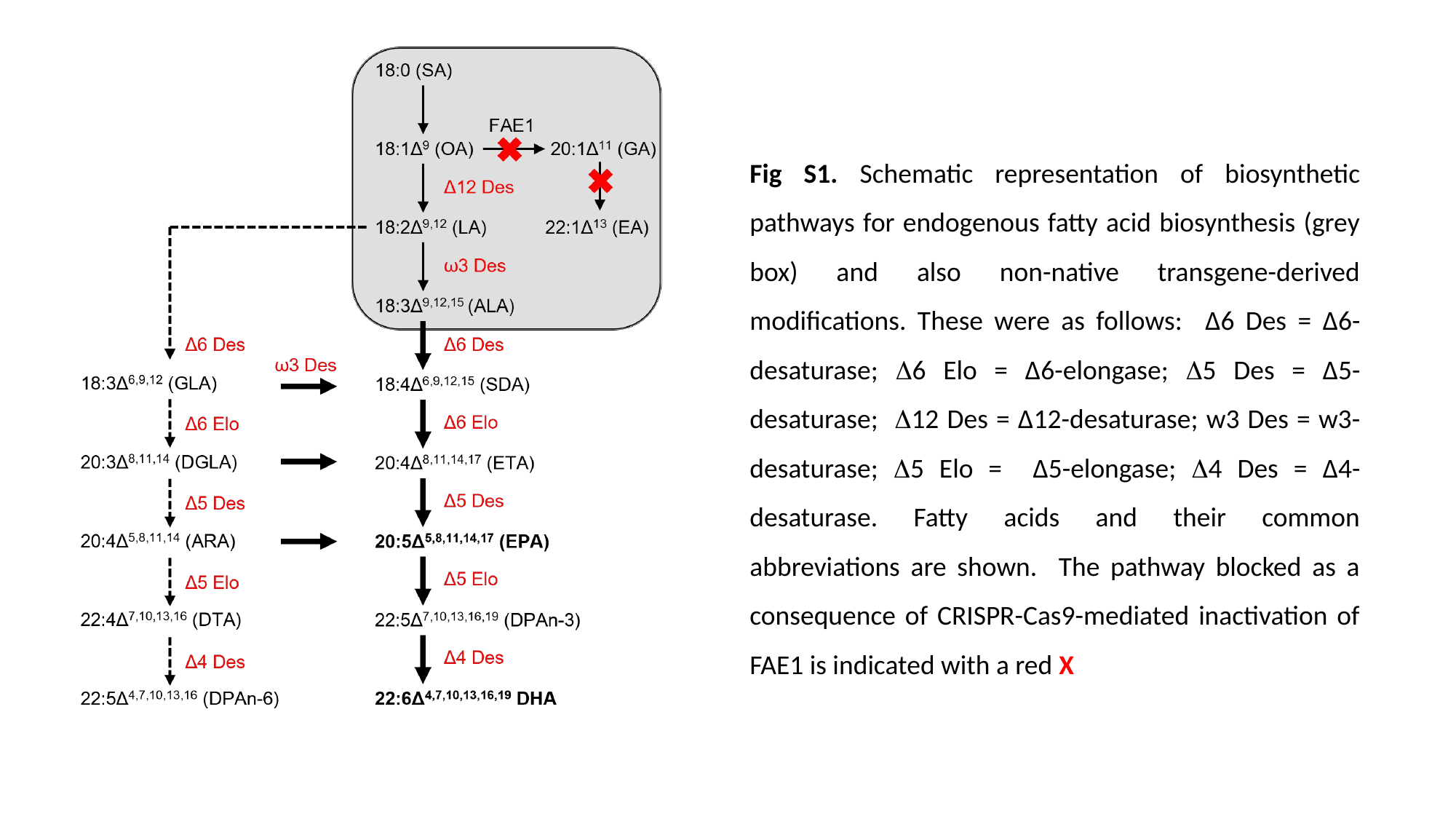

Fig S1. Schematic representation of biosynthetic pathways for endogenous fatty acid biosynthesis (grey box) and also non-native transgene-derived modifications. These were as follows: Δ6 Des = Δ6-desaturase; D6 Elo = Δ6-elongase; D5 Des = Δ5-desaturase; D12 Des = Δ12-desaturase; w3 Des = w3-desaturase; D5 Elo = Δ5-elongase; D4 Des = Δ4-desaturase. Fatty acids and their common abbreviations are shown. The pathway blocked as a consequence of CRISPR-Cas9-mediated inactivation of FAE1 is indicated with a red X

## Slide 2
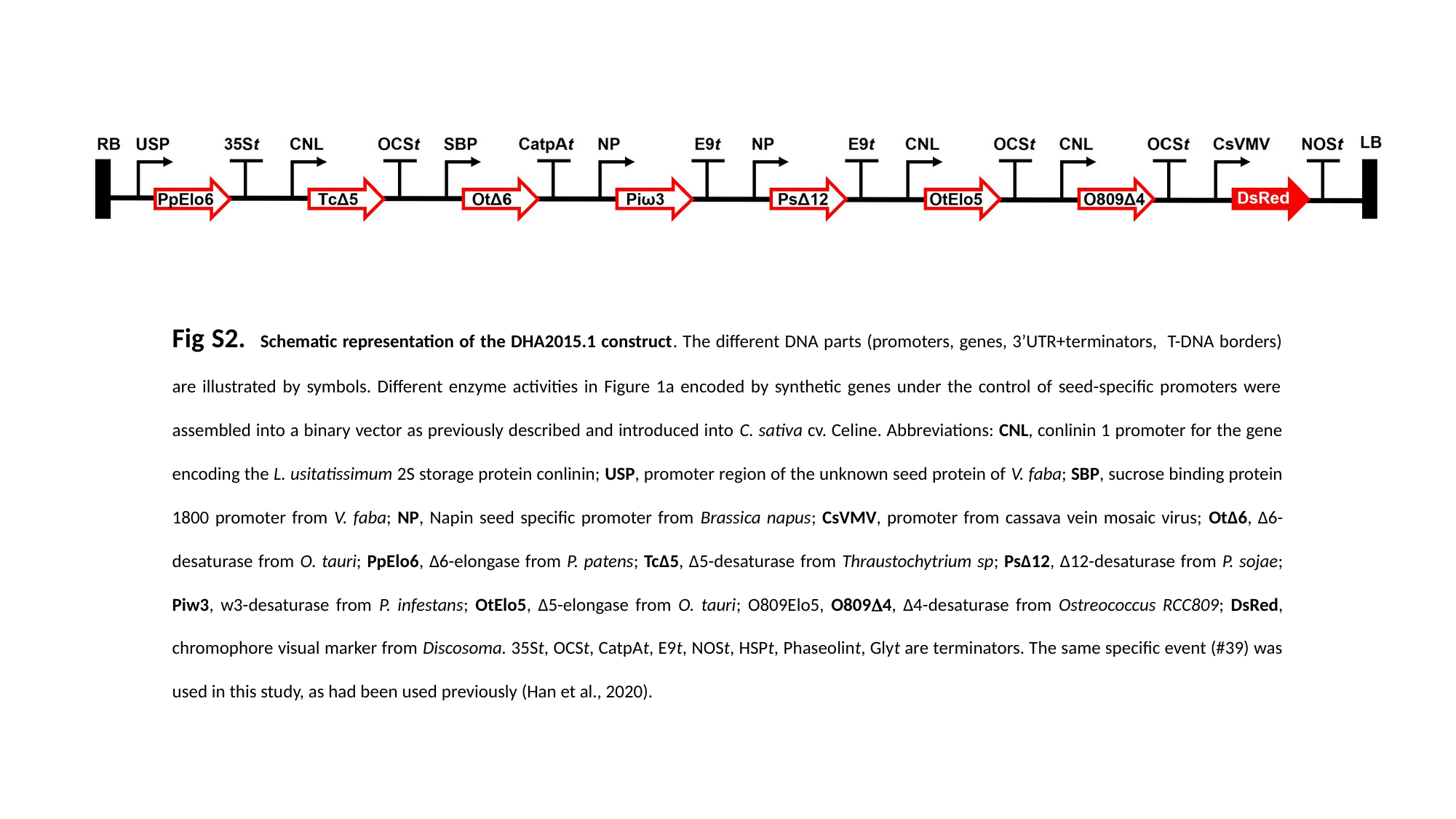

Fig S2. Schematic representation of the DHA2015.1 construct. The different DNA parts (promoters, genes, 3’UTR+terminators, T-DNA borders) are illustrated by symbols. Different enzyme activities in Figure 1a encoded by synthetic genes under the control of seed-specific promoters were assembled into a binary vector as previously described and introduced into C. sativa cv. Celine. Abbreviations: CNL, conlinin 1 promoter for the gene encoding the L. usitatissimum 2S storage protein conlinin; USP, promoter region of the unknown seed protein of V. faba; SBP, sucrose binding protein 1800 promoter from V. faba; NP, Napin seed specific promoter from Brassica napus; CsVMV, promoter from cassava vein mosaic virus; OtΔ6, Δ6-desaturase from O. tauri; PpElo6, Δ6-elongase from P. patens; TcΔ5, Δ5-desaturase from Thraustochytrium sp; PsΔ12, Δ12-desaturase from P. sojae; Piw3, w3-desaturase from P. infestans; OtElo5, Δ5-elongase from O. tauri; O809Elo5, O809D4, Δ4-desaturase from Ostreococcus RCC809; DsRed, chromophore visual marker from Discosoma. 35St, OCSt, CatpAt, E9t, NOSt, HSPt, Phaseolint, Glyt are terminators. The same specific event (#39) was used in this study, as had been used previously (Han et al., 2020).

## Slide 3
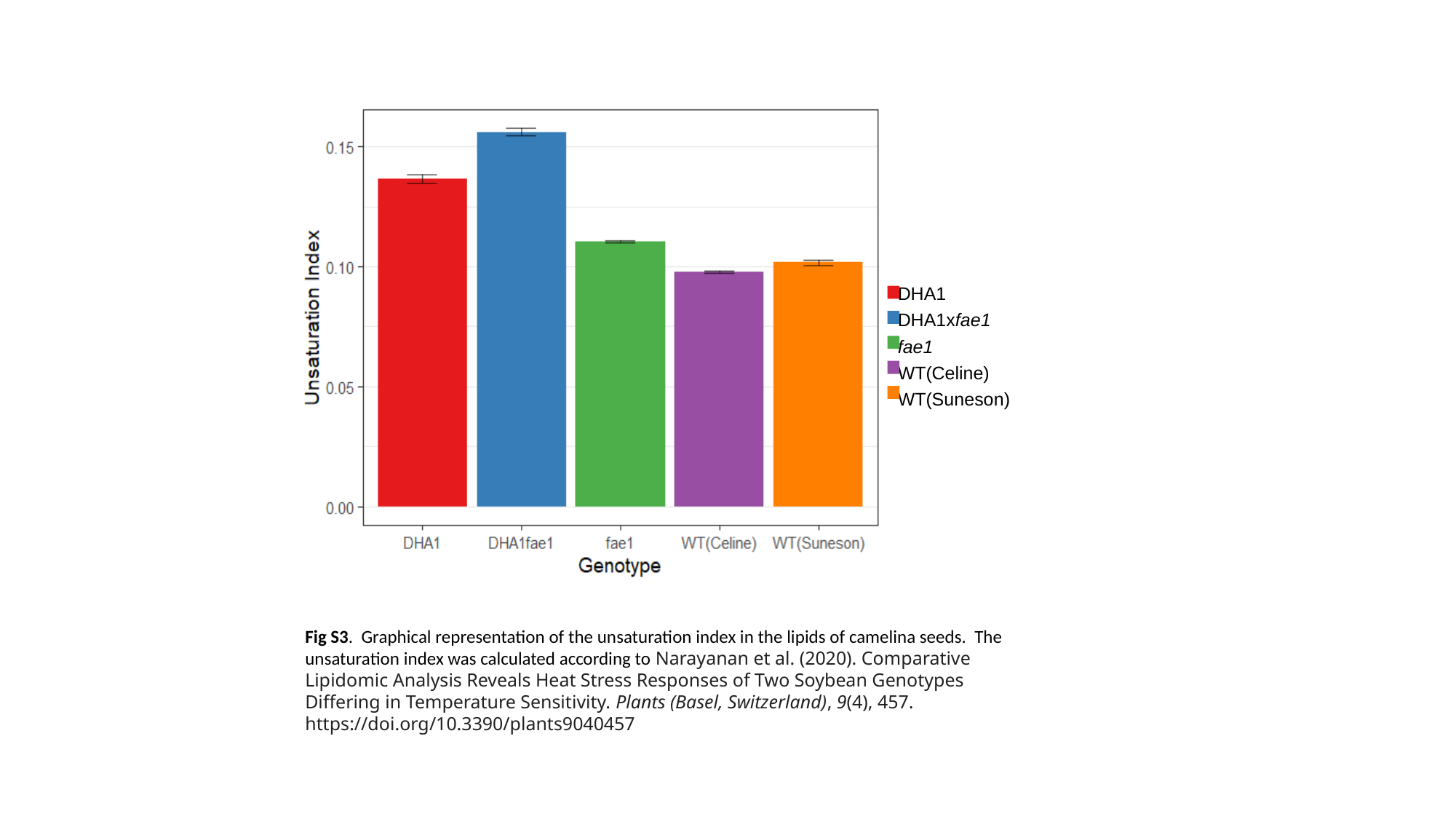

DHA1
DHA1xfae1
fae1
WT(Celine)
WT(Suneson)
Fig S3. Graphical representation of the unsaturation index in the lipids of camelina seeds. The unsaturation index was calculated according to Narayanan et al. (2020). Comparative Lipidomic Analysis Reveals Heat Stress Responses of Two Soybean Genotypes Differing in Temperature Sensitivity. Plants (Basel, Switzerland), 9(4), 457. https://doi.org/10.3390/plants9040457

## Slide 4
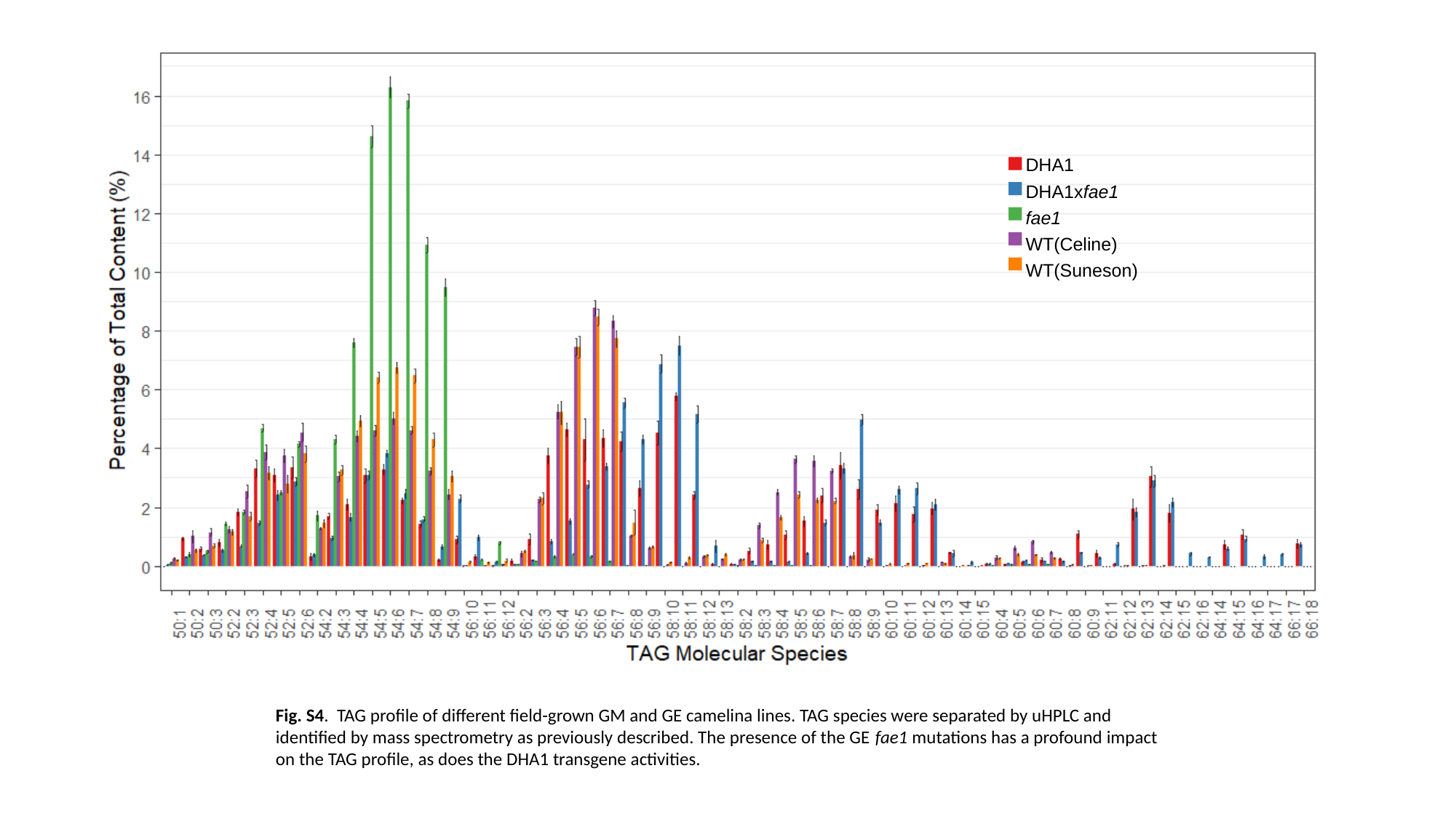

DHA1
DHA1xfae1
fae1
WT(Celine)
WT(Suneson)
Fig. S4. TAG profile of different field-grown GM and GE camelina lines. TAG species were separated by uHPLC and identified by mass spectrometry as previously described. The presence of the GE fae1 mutations has a profound impact on the TAG profile, as does the DHA1 transgene activities.
